# Downregulation of the Protein C Signaling System Is Associated with COVID-19 Hypercoagulability—A Single-Cell Transcriptomics Analysis

**DOI:** 10.3390/v14122753

**Published:** 2022-12-09

**Authors:** Bruna Rafaela Santos Silva, Carlos Poblete Jara, Davi Sidarta-Oliveira, Licio A. Velloso, William H. Velander, Eliana P. Araújo

**Affiliations:** 1 Nursing School, University of Campinas, Tessalia Vieira de Camargo, 126, Campinas 13084-970, Brazil; 2 Laboratory of Cell Signalling, Obesity and Comorbidities Center, OCRC, University of Campinas, Carl Von Linnaeus, s/n, Campinas 13084-864, Brazil; 3 Department of Chemical and Biomolecular Engineering, University of Nebraska, Lincoln, NE 68588-0643, USA; 4 School of Medical Sciences, University of Campinas, Tessalia Vieira de Camargo, 126, Campinas 13083-887, Brazil

**Keywords:** antigen-presenting cell, computational biology, blood coagulation disorders, endothelial cells, SARS-CoV-2

## Abstract

Because of the interface between coagulation and the immune response, it is expected that COVID-19-associated coagulopathy occurs via activated protein C signaling. The objective was to explore putative changes in the expression of the protein C signaling network in the liver, peripheral blood mononuclear cells, and nasal epithelium of patients with COVID-19. Single-cell RNA-sequencing data from patients with COVID-19 and healthy subjects were obtained from the COVID-19 Cell Atlas database. A functional protein–protein interaction network was constructed for the protein C gene. Patients with COVID-19 showed downregulation of protein C and components of the downstream protein C signaling cascade. The percentage of hepatocytes expressing protein C was lower. Part of the liver cell clusters expressing protein C presented increased expression of *ACE2*. In PBMC, there was increased *ACE2*, inflammatory, and pro-coagulation transcripts. In the nasal epithelium, *PROC*, *ACE2,* and *PROS1* were expressed by the ciliated cell cluster, revealing co-expression of ACE-2 with transcripts encoding proteins belonging to the coagulation and immune system interface. Finally, there was upregulation of coagulation factor 3 transcript in the liver and PBMC. Protein C could play a mechanistic role in the hypercoagulability syndrome affecting patients with severe COVID-19.

## 1. Introduction

Coronavirus disease 2019 (COVID-19)-associated coagulopathy is defined as a systemic activation of coagulation that is characterized by endotheliopathy, with activation of the innate and adaptive immune response and the complement system [1]. Indeed, blood coagulation disorder was described during the 2003 outbreak of severe acute respiratory syndrome coronavirus (SARS-CoV) infection [2]. In affected patients, there is acute-phase diffuse alveolar damage with the formation of hyaline membranes and a fibrin-rich exudate in the alveolar space with hemostatic alterations [3]. In addition to alveolar destruction and the presence of blood exudate, the capillaries present dilation, congestion, mononuclear cell infiltration, and thrombosis [4]. There are macroscopic thrombi and fibrin microthrombi inside alveolar capillaries in patients with severe COVID-19. These changes suggest that effective therapies should not only target the virus itself but also the thrombotic and angiopathic effects resulting from the infection [5]. The prothrombotic status of critical patients with extrapulmonary thrombotic manifestations [6,7,8] implies that severe COVID-19 is a multisystem syndrome. First, the disease affects the vascular endothelium [7,8], with a reduction in the fibrinolytic response that potentiates microthrombus events, which then contribute to the progression of the disease toward the fibroproliferative phase.

Currently, there is an ongoing effort to develop strategies that could improve the pharmacological treatment of COVID-19 complications. This has led to the testing of antiviral agents [9], convalescent plasma [10], monoclonal antibodies [11], steroids [12], and inhibitors of bradykinin [13]. In general, they have failed to protect patients from the development of serious thrombotic complications. Even vaccination, which has reduced the number of cases in the United States [14], has not proved to be effective in reducing the risk of thrombotic complications in patients who develop severe COVID-19 [15]. Based on the functional interface between coagulation and the immune response [16], researchers have hypothesized that targeting coagulation pathways ameliorates the dysregulated immune response observed in patients with COVID-19. In fact, anticoagulant treatment in patients with COVID-19 has proved to be effective in critical stages of the disease [17]; however, there is still no consensus about the mechanisms underlying this threatening condition [18]. In this study, we explored the hypothesis that COVID-19 is related to the dysregulation of tissue factors and to the downregulation of the innate anticoagulation dependent on the activated protein C (APC) signaling.

**Figure 1:**
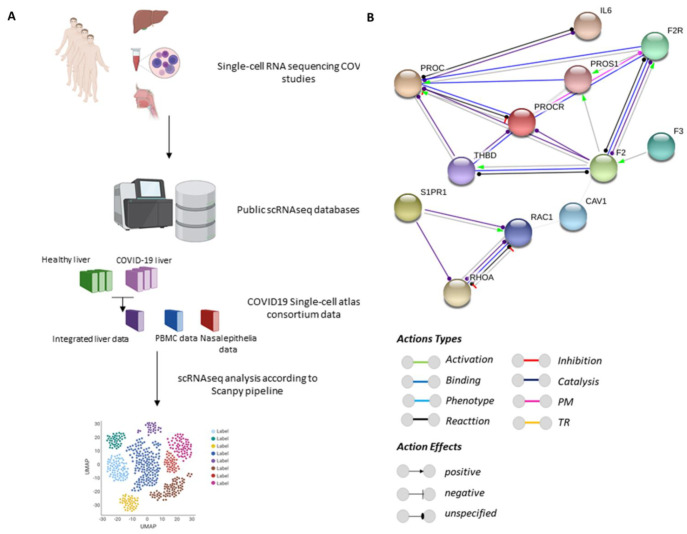
Experimental design and protein–protein interaction network. (**A**). Public data from single-cell RNA sequencing (scRNA-seq) studies on the human tissues (liver, peripheral blood mononuclear cells (PBMC), and nasal epithelia) were retrieved from the website portal www.COVID-19cellatlas.org accessed on 16 April 2022 and Gene Expression Omnibus (GEO) accession numbers GSE171668 and GSE115469. (**B**). The PROC interactome was retrieved with the string-db data mining toolkit (https://version-11-0.string-db.org/cgi/network.pl?taskId=lu4XDH7F7cOZ, accessed on 16 April 2022). The sources used for functional interactions were *neighborhood*, *experiments*, *gene fusion*, *databases*, *co-occurrence* and *co-expression*, and visualized by the ‘molecular actions’. The network nodes represent proteins and the edges represent protein–protein associations. The edges indicate the known molecular action of a protein node relative to another protein node. PM: post-translational modification; TR: transcriptional regulation.

Protein C (PC) synthesis occurs mainly in hepatocytes; it is then released into the bloodstream as a zymogen [19]. Previous studies have shown that PC is expressed in human tissues such as the male reproductive organs [20], kidneys, and urinary bladder [21]. In addition, recombinant human PC has been produced in the porcine mammary gland [22]. APC exerts cytoprotective effects via anti-inflammatory and anti-apoptotic activities, as well as protecting endothelial barrier function [23]. Zymogen PC is present in the blood and is proteolytically activated by the thrombin-thrombomodulin (TM) complex on the surface of endothelial cells. This activation occurs in the presence of the membrane receptor endothelial protein C receptor (EPCR). EPCR facilitates the interaction between PC with the TM complex, therefore increasing the generation of APC [24]. When binding to EPCR, APC exerts most of its cytoprotective effects through the cleavage of protease-activated receptor-1 (PAR-1) [25]. PAR-1 cleavage in the N-terminal exodomain triggers the activation of endothelial cytoprotective molecules such as Rac1. Additionally, cleavage of PAR-1 triggers the inhibition of RhoA, a small GTPase that regulates endothelial barrier permeability [26]. The interface between PC and the inflammatory system occurs mostly through interleukin 6 (IL-6), which is secreted by endothelial cells [23], affecting the immune response, cell proliferation and differentiation [27]. Here, we used bioinformatics to analyse single-cell transcriptomes from patients with COVID-19 (Figure 1A). We explored components of the PC signaling system. We identified changes in the expression of the PC signaling network in the liver, peripheral blood mononuclear cells (PBMC) and nasal epithelium of patients with COVID-19. This descriptive study suggests the PC system is involved in the local and systemic derangement of haemostatic mechanisms in COVID-19.

## 2. Methods

### 2.1. Protein–Protein Interaction Networks

Protein functional interaction networks for PC signaling proteins were prepared using the STRING v11 database, a software toolkit that performs data mining on a large number of databases and on individually published high-throughput datasets to build putative protein interactome maps. The association evidence in the STRING database comes from independent sources (‘channels’): genomic context information (neighborhood, fusion, gene co-occurrence), co-expression, text-mining, biochemical/genetic data (‘experiments’), and previously curated pathways and protein complex datasets (‘databases’) [28]. The default settings for functional interaction networks were queried for PC signaling proteins in *Homo sapiens*. The association’s sources used for functional interactions were *neighborhood*, *experiments*, *gene fusion*, *databases*, *co-occurrence,* and *co-expression*, and visualized by the *molecular actions*. The network nodes represent proteins and edges represent protein–protein associations. A permalink webpage of the PROC protein–protein interaction network is accessible at https://version-11-0.string-db.org/cgi/network.pl?networkId=1bGTXH59glgq, accessed on 16 April 2022.

### 2.2. Analysis of Single-Cell RNA-Sequencing (scRNA-seq) Data

For the expression of receptors and PC-related genes, datasets were retrieved from published studies containing data from multiple human tissues, including the liver [29,30], nasal epithelium [31,32], and peripheral blood [32,33]. These datasets are available and can be visualized and assessed through the website portal www.COVID-19cellatlas.org, accessed on 1 February 2022 [32]. In addition, a folder within all data analyzed is available on https://drive.google.com/drive/folders/12nmcAwphN8Zl8as8qgFiiJBafVcbsK6o?usp=sharing, accessed on 20 October 2022. The processed .h5ad files were loaded by using *read_h5ad* and matrix plots were illustrated using *sc.pl.matrixplot* in Scanpy 1.7.2, which is a model for single-cell analysis in Python [34]. For the scRNA-seq analysis of liver cells in patients with COVID-19, a dataset reported by Delorey et al. [29] was retrieved from Gene Expression Omnibus (GEO) with accession number GSE171668; control patients were retrieved from the GEO accession number GSE115469, a dataset reported by MacParland et al. [30]. The raw data with the .h5 format were loaded for analysis through the toolkit Scanpy 1.7.2 in Python igraph 0.9.6. For each sample, cells were filtered out if they contained fewer than 200 genes, more than 1500 genes or if mitochondrial genes were >15% of the total. The remaining cells were integrated into a gene-barcode matrix and then normalized and log transformed. The highly variable genes were identified by using the *pp.highly_variable_genes* function for the downstream principal component analysis (PCA) [35]. For the neighborhood graph of cells, the PCA representation of the data matrix was used and clustering was performed by Leiden graph-clustering method [36]. We chose the top 50 principal components for the Uniform Manifold Approximation and Projection (UMAP) and graph-based clustering. The cell type identity was manually annotated and the differentially expressed genes (DEG) for a cluster and all remaining cells calculated by the two-sided Wilcoxon rank-sum test [37]. The marker genes were then ranked by their log fold change of expression in particular cell types. The Supplementary Material for all tissues analyzed is available with marker genes for the cells type (Supplementary Tables S1–S3).

### 2.3. Statistical Analyses

The DEGs of patients with COVID-19 compared with healthy controls were determined with Scanpy’s implementation of the two-sided Wilcoxon rank-sum test [37]. The *p_val_cutoff* = 0.05 and *logfc_cutoff* = 0.1 were established by the *Scanpy utils* function.

### 2.4. Data Availability

Publicly available processed data used in this study with identified metadata and embeddings are available for download from the COVID-19 Cell Atlas (https://www.COVID-19cellatlas.org/, accessed on 1 February 2022) [32] hosted by the Wellcome Sanger Institute. The raw sequencing data from Delorey et al. [29] and MacParland et al. [30] are available at the NCBI Gene Expression Omnibus, accession numbers GSE171668 and GSE115469, respectively, and also available to download at https://drive.google.com/drive/folders/12nmcAwphN8Zl8as8qgFiiJBafVcbsK6o?usp=sharing, accessed on 20 October 2022.

### 2.5. Code Availability

All scripts used for data analysis are available from GitHub (https://github.com/OCRC/BRSilva_2022, accessed on 20 October 2022).

### 2.6. Generation of Diagrams and Illustrations

A licensed version of BioRender was employed to generate the diagrams present in most figures.

## 3. Results

### 3.1. PROC Interacts with Proteins Belonging to Barrier Integrity and the Inflammatory and Coagulation Systems

The protein–protein interaction network (Figure 1B) shows the reactions and binding interactions between PC (PROC) and its receptor, EPCR (PROCR), and the effects (unspecified, negative, and positive). PROC has a binding interaction with protein S (PROS1) and protease-activated receptor 1 (F2R), evidenced by the Experimental/Biochemical Association in Curated Databases. In addition, PROCR has a post-translational modification interaction with F2R with an unspecified effect. THBD shows a catalytic interaction with an unspecified effect with PROC and PROCR and a mutual interaction with an unspecified effect with F2. F2 shows binding, reaction, and catalytic interactions with an unspecified and positive effect with F2R and a positive effect with PROS1. PC also interacts with IL-6 through a reaction and catalytic interaction with an unspecified effect. F3 shows a positive effect with F2. Finally, S1PR1 interacts by catalysis with RAC1 and RHOA, with an unspecified and positive effect. RAC1 and RHOA present a mutual negative effect and interact by binding, catalysis, and reaction interactions with an unspecified effect.

### 3.2. PC pathway Landscape at the Single-Cell Level in Tissues from Patients with COVID-19

**Figure 2:**
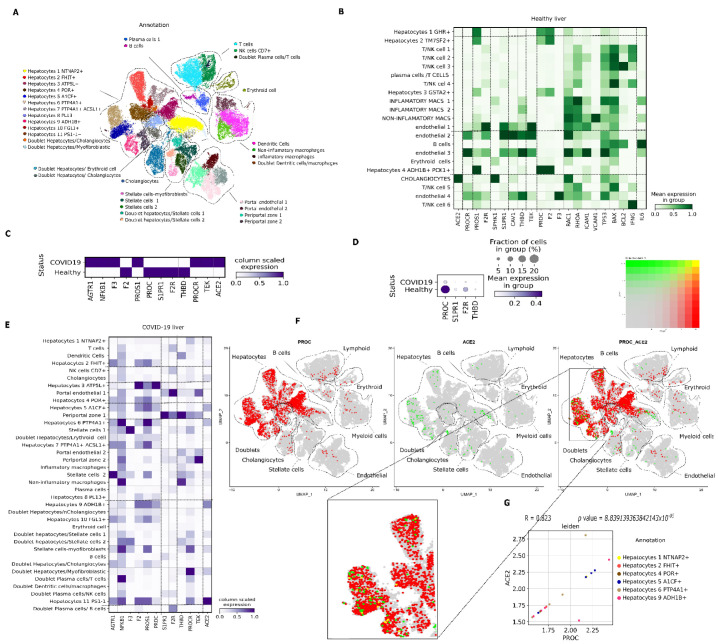
Expression of genes involved in activated protein C (APC) signaling in the liver from healthy individuals and patients with coronavirus disease 2019 (COVID-19) based on single-cell transcriptomics. (**A**). Uniform Manifold Approximation and Projection (UMAP) embedding of liver cells via single-cell RNA sequencing (scRNA-Seq) droplet-based single-cell platform, showing single liver parenchymal cells grouped into 36 clusters. (**B**). Matrix plot showing expression of genes related to APC signaling according to the cell type in a healthy liver. (**C**). Matrix plot showing expression of genes related to APC signaling in patients with COVID-19 versus healthy individuals. (**D**). Dot plot representation of downregulated genes in patients with COVID-19. (**E**). Matrix plot showing expression of genes related to APC signaling according to the cell type in an autopsy liver sample from a patient with COVID-19. (**F**). Visualization of co-expression (yellow) by color overlap. *PROC* (red) is barely co-expressed with *ACE2* (green) in hepatocyte clusters. (**G**). Spearman’s correlation of subset of cells that co-expressed *PROC* and *ACE2*.

**Figure 3:**
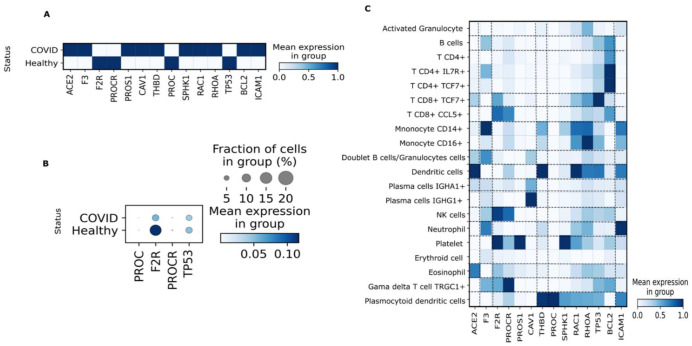
Expression of genes involved in activated protein C (APC) signaling in human peripheral blood mononuclear cells (PBMC) from patients with coronavirus disease 2019 (COVID-19) based on single-cell transcriptomics. (**A**). Matrix plot showing expression of genes related to APC signaling in patients with COVID-19 versus healthy individuals. (**B**). Dot plot representation of downregulated genes in patients with COVID-19. (**C**). Matrix plot showing expression of the main genes related to APC signaling according to cell types.

**Figure 4:**
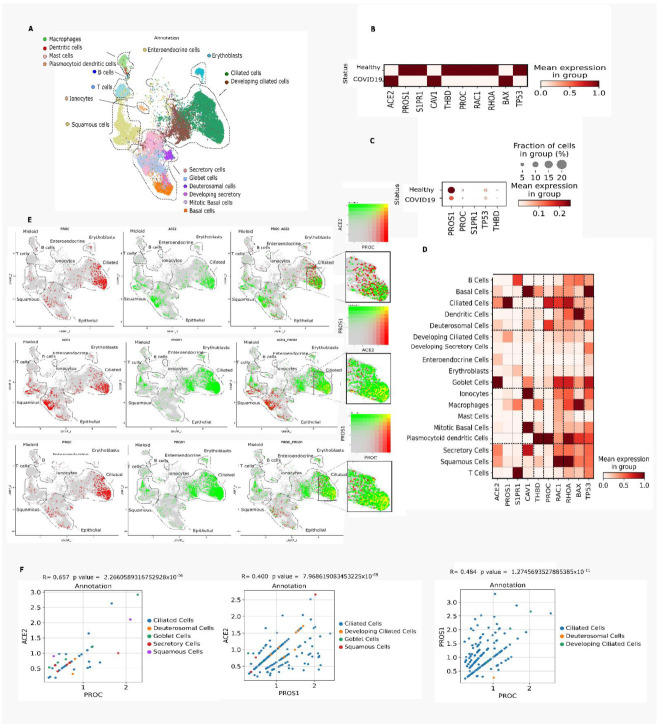
Expression of genes involved in activated protein C (APC) signaling in nasal epithelial cells from patients with coronavirus disease 2019 (COVID-19) based on single-cell transcriptomics. (**A**). Uniform manifold approximation and projection (UMAP) embedding of nasal epithelial cells via single-cell RNA sequencing (scRNA-Seq) droplet-based single-cell platform grouped into 18 clusters. (**B**). Matrix plot showing expression of genes related to APC signaling in patients with COVID-19 versus uninfected individuals. (**C**). Dot plot representation of downregulated genes in patients with COVID-19. (**D**). Matrix plot showing expression of the main genes related to APC signaling according to cell types retrieved from Nasal epithelial samples. (**E**). Visualization of co-expression (yellow) by color overlap. *PROC* (red) is barely co-expressed with *ACE2* (green) in ciliated cell clusters. *ACE2* (red) is co-expressed with *PROS1* (green) in ciliated cell clusters. *PROC* (red) is co-expressed with *PROS1* (green) in ciliated cell clusters. (**F**). Spearman’s correlation of a subset of cells that expressed *PROC*, *PROS1,* and *ACE2*.

To study transcriptional changes in PC signaling in patients with COVID-19, gene expression of PC signaling-associated genes was explored in liver samples (Figure 2A–G), PBMC (Figure 3A–C), and nasal epithelia (Figure 4A–F) from patients with severe COVID-19 compared with healthy controls.

Differentially expressed genes between patients with COVID-19 and healthy controls were determined with Scanpy’s implementation of the two-sided Wilcoxon rank-sum test. Genes lacking a value (denoted as ‘-’) did not reach the logFC threshold of 0.1. COVID-19 %: percentage of cells in the COVID-19 group that expresses the gene. Healthy %: percentage of cells in the healthy group that expresses the gene. Abbreviations—logFC: log fold change; PBMC: peripheral blood mononuclear cells.

*Liver.* PC synthesis occurs mainly in hepatocytes, and it is released into the bloodstream as a zymogen [19]. First, we determined the expression of the *PROC* gene in healthy liver parenchyma and then compared the expression to samples collected from autopsies of patients who died because of severe COVID-19 complications. *PROC* was expressed throughout the liver parenchyma of healthy individuals, particularly in hepatocyte 4 (ADH1B^+^/PCK1^+^), which displayed the highest expression (Figure 2B). *ACE2* expression was restricted to cholangiocytes in the healthy liver; the main receptors involved in PC signaling—*PROCR*, *F2R*, *S1PR1*, *THBD,* and *TEK*—were detected in endothelial cells (Figure 2B). In liver autopsy samples from patients who died because of COVID-19 complications, PC activation signaling receptors were significantly decreased compared with control as follows: 2.7-fold for *S1PR1*, 2.5-fold for *F2R* and 1.7-fold for *THBD* (*p* < 0.001), and less than 5% of cells expressed these genes (Table 1; Figure 2C,D). Conversely, liver samples from patients with COVID-19 presented a significant 1.9-fold increase in *AGTR1*, a 1.8-fold increase in *NFKB1,* and a 1.2-fold increase in *F3* (*p* < 0.001) (Table 1; Figure 2C). Additionally, there was a significant 2.192-fold decrease in *PROC* in liver samples from individuals who died from COVID-19 complications (*p* < 0.001) (Table 1; Figure 2C). The relative number of hepatocytes from patients with COVID-19 expressing *PROC* was lower (about 5%) than in healthy individuals (about 20%) (Table 1; Figure 2D). The highest expression of *PROC* occurred in hepatocyte 3 (ATP5L^+^), followed by hepatocytes 9 (ADH1B^+^), 5 (A1CF^+^), and 6 (PTP4A1^+^) (Figure 2E). *ACE2* gene expression was 2.868-fold higher in patients with COVID-19 (*p* = 0.000) (Table 1), and the expression was no longer restricted to cholangiocytes (as in the healthy liver) but extended to the liver parenchyma. *ACE2* was expressed by hepatocytes 9 (ADH1B^+^), 5 (A1CF^+^), 6 (PTP4A1^+^), and 2 (FHIT^+^) with low *PROC* co-expression (Figure 2E,F). Moreover, there was a very strong association between *ACE2* and *PROC* gene expression in samples from patients with COVID-19 between the cells that co-expressed these two genes (Figure 2G).

### 3.3. PBMC

Considering the effects of APC on the inflammatory response that leads to reduced immune cell nuclear factor κB (NF-κB)-related protein syntheses [38], we determined the expression of genes related to the PC signaling pathway in PBMC of patients with COVID-19 within 10 days of the onset of symptoms. In COVID-19 samples, there were significant decreases in the following genes compared with healthy individuals: 1.127-fold for *F2R*, 1.097-fold for *PROCR*, 1.040-fold for *PROC,* and 0.193-fold for *TP53* (*p* < 0.05) (<10% of PBMC expressed these genes compared with healthy controls) (Table 1; Figure 3A,B). Conversely, there was a significant 2.943-fold increase in *ACE2* expression and significantly altered expression of pro-inflammatory and pro-coagulation genes, including 1.306-fold for *F3* and 0.548-fold for *ICAM1* (*p* < 0.05) in patients with COVID-19 compared with healthy controls (Table 1; Figure 3A). *ACE2* expression was detected in dendritic cells, eosinophils, IGHA1^+^ plasma cells, and CD8^+^ TCF7^+^ T cells. The *F3* gene was detected in CD14^+^ monocytes, neutrophils, TRGC1^+^ gamma-delta T cells, CD4^+^ T cells, and B cells. *ICAM1* expression was detected in neutrophils, dendritic cells, CD14^+^ monocytes, plasmacytoid dendritic cells, and CD16^+^ monocytes (Figure 3C). Moreover, there was a heterogeneous expression of PC activation receptors in other cell populations, such as platelets, natural killer (NK) cells, CD8^+^ CCL5^+^ T cells, TRGC1^+^ gamma-delta T cells, plasmacytoid dendritic cells, dendritic cells and IGHG1^+^ plasma cells, each expressing distinct PC activation receptors including *F2R*, *PROCR*, *THBD,* and *CAV1* (Figure 3C).

### 3.4. Nasal Epithelium

In the early stages of COVID-19, the upper respiratory tract is the primary target for severe acute respiratory syndrome coronavirus 2 (SARS-CoV-2) replication [39]. Hence, we looked for transcriptional changes in the PC activation and signaling-associated genes in nasal epithelial cells of patients with COVID-19 who had been hospitalized for 1–3 days. In COVID-19 samples, there was a significant decrease in the following genes compared with healthy subjects: 0.985-fold for *PROS1*, 1.108-fold for *S1PR1*, 1.323-fold for *THBD*, 0.502-fold for *PROC*, 0.767-fold for *RAC1*, 0.841-fold for *RHOA,* and 0.207-fold for *TP53* (*p* < 0.05) (Table 1; Figure 4B). Less than 5% of nasal epithelial cells from patients with COVID-19 expressed *PROS1* compared with healthy controls (Figure 4C). *PROC* expression was detected in plasmacytoid dendritic, ciliated, deuterosomal, basal, and goblet cells (Figure 4D). Basal cells expressed the most APC signaling-associated genes as well as those regulated in response to APC activation (*CAV1*, *THBD*, *PROC*, *RAC1*, *RHOA*, *BAX,* and *TP53*) (Figure 4D). Analysis of nasal epithelium showed that the *PROC*, *ACE2*, and *PROS1* genes were expressed by the same ciliated cells, showing *PROS1*–*ACE2* and *PROC*–*PROS1* co-expression (Figure 4E,F). In addition, there was a strong association (*p* < 0.05) between *ACE2* and *PROC* gene expression in ciliated cells from patients with COVID-19 (Figure 4F).

## 4. Discussion

The inflammation and coagulation axis has emerged as one of the main mechanisms controlling the host response to invading microorganisms, and the imbalance in this mechanism is responsible for the poor prognosis in patients with severe sepsis, including the risk of death [40]. Sepsis is a complex syndrome that results from infection [41] mainly by bacteria; however, studies suggest that the prevalence of viral sepsis is often underestimated [42,43]. Respiratory viral sepsis is a highly heterogeneous and multifaceted syndrome that can cause extrapulmonary organ dysfunction resulting from respiratory virus infections [41]. Acute kidney and cardiac injuries have been reported in influenza virus infection; infection caused by Middle East respiratory syndrome-related coronavirus (MERS-CoV) is related to acute kidney injury and thrombocytopenia. Hepatic dysfunction has been reported for respiratory syncytial virus infection. Furthermore, among fatal cases of SARS-CoV infection, high viral loads in the intestine and liver and moderate viral loads in the kidney have been reported [44,45,46,47]. Since the initial cases of COVID-19, there have been patients with a severe form of the disease, including sepsis, who have died as a result of the infection. They showed decreased platelet counts and elevated bilirubin and creatinine, reflecting clear signs of clotting disorder in addition to liver and kidney dysfunction [48,49]. In this study, we used the opportunity to assess several different vascularized tissues to detail the expression of one of the main pathways involved in hemostasis and endogenous immune responses in patients with severe COVID-19. We identified that the PC system may be involved in local and systemic derangement of hemostatic mechanisms in COVID-19.

Complications related to hyper-inflammation have been described in critical patients with COVID-19 [50]. Angiotensin-converting enzyme 2 (ACE2) is an important vasoconstriction enzyme that regulates vascular tone through its effector peptide angiotensin 1–7 (Ang (1–7)), and receptor Mas1 induces vasodilation and attenuates Ang II-induced vasoconstriction [51,52]. The coagulation cascade is intimately tied to ACE2 responses via the FXII contact activation pathway [53,54]. SARS-CoV-2 may contribute to Ang II-induced thrombosis by an imbalance in ACE2 levels, causing angiotensin-induced coagulopathy, with a high risk of thrombosis, comprising platelet activation, thrombin generation, and endothelial damage via the type 1 angiotensin II receptor [54]. Previous studies have shown that modulation of ACE2 is correlated to the time of infection and the tissue affected [55]. We found that patients with COVID-19 had elevated *ACE2* messenger RNA (mRNA) levels throughout the liver parenchyma and decreased *PROC* expression. In silico analysis of patients with COVID-19 showed that high *ACE2* expression enhances the expression of genes involved in replication, assembly and prolonging the virus life cycle [55]. Here, we showed that in COVID-19 samples, *ACE2* was also expressed by the hepatocytes responsible for *PROC* expression. We suggest that the lower *PROC* mRNA levels found in the liver may be related to *ACE2* expression in those hepatocytes.

The renin-angiotensin system (RAS) has been identified as a potential driver of host pulmonary and systemic immune responses, where the physiological balance between pro-inflammatory, vasoconstrictive, and pro-fibrotic effects of angiotensinogen through the angiotensin II receptor-like receptor 1 (AGTR1) are maintained in healthy subjects by equilibrium with AGTR2 and by the engagement of the Mas receptor by Ang (1–7) [52,56]. Here, we observed increased hepatic expression of *F3*, *AGTR1,* and *NFKB1* in COVID-19 individuals. Previous studies have shown that hepatocyte tissue factor contributes to the hypercoagulable state in a mouse model of chronic liver injury [57]. After binding AngII to AT1R, the receptor mediates several important systemic effects in the liver, including vascular, proliferative, and inflammatory reactions [58]. In addition, injury-induced activation of hepatic NF-κB is observed in a variety of non-parenchymal and parenchymal liver cells, indicating that NF-κB plays a central role in coordinating the inflammatory response and wound healing by stimulating gene transcription in several key cellular players [59]. A computer model identified potentially beneficial drugs for critically COVID-19 patients that act by inhibiting the tumor necrosis factor (TNF)-induced NFkB1 signaling pathway [60], which is inhibited by APC activity [61].

The change in transcription of *PROC* and the activator receptors *PROCR*, *FR2,* and *THBD* in patients with COVID-19 suggests impaired cytoprotection and progression to the hypercoagulable state [62,63]. This progression starts with pathogen-induced cellular stress, a concurrent increase in tissue factor expression, and ensuing vascular cell death. Tissue factor has been shown to be upregulated in bronchial epithelial cells infected with SARS-CoV-2 [63]. The chief role of PC is to counteract the cell stress and apoptosis induced by cytokines and tissue factors released as part of the inflammatory response of bacterial and viral sepsis. Indeed, recent studies have shown that decreased plasma PC levels in patients with COVID-19 at hospital admission were associated with a worse clinical prognosis [64,65]. Our analysis revealed low *PROC* expression in the nasal epithelium during days 1–3 of hospitalization and in PBMC within 10 days of symptom onset. However, in the analysis of patients admitted to the intensive care unit (ICU), two independent cohorts revealed high PC plasma levels [65] but low functional activity [66]. Here, we explored *PROC* mRNA expression in patients who did not overcome the infection. We observed that the expression of hepatic *PROC* was decreased in these patients.

We found that *PROC*, *S1PR1,* and *THBD* were downregulated in the nasal epithelium of patients with COVID-19. Human nasal respiratory epithelial cells are primary targets for SARS-CoV-2 replication in the early stages of COVID-19 infection [16,39]. Compared with the oral squamous epithelium, viral tropism is restricted to the cells of the nasal epithelium [67] because they strongly express SARS-CoV-2 entry factors along with innate immunity genes, highlighting the role of these cells in early viral infection, dissemination, and elimination. Our analyses revealed that these cells also harbor intrinsic anticoagulation mechanisms and express genes correlated with inflammation that co-express with *ACE2* (the virus receptor) in nasal ciliated cells. The co-expression between *ACE2* and genes that facilitate the infection and that may contribute to the evolution of disease has been previously described in corneal tissue [68].

Previous studies have shown important dysregulation in the immune system of patients with severe COVID-19 [69,70]. Our PBMC analyses showed lower expression of *PROC*, *F2R,* and *PROCR* in patients with COVID-19 compared with controls. These genes are already known to be key to the activation and signaling of PC [71]. In addition, immature neutrophils and immunosuppressant cells in patients with COVID-19, followed by high levels of inflammatory cytokines, could be linked to PC inhibition, leading to the activation of the coagulation cascade. In addition, inflammatory factors can increase the formation of plasminogen activator inhibitor-1 (PAI-1), the synthesis of which increases during COVID-19 [72], and prevent fibrinolysis, thus promoting coagulation and even the formation of microthrombi.

**Figure 5:**
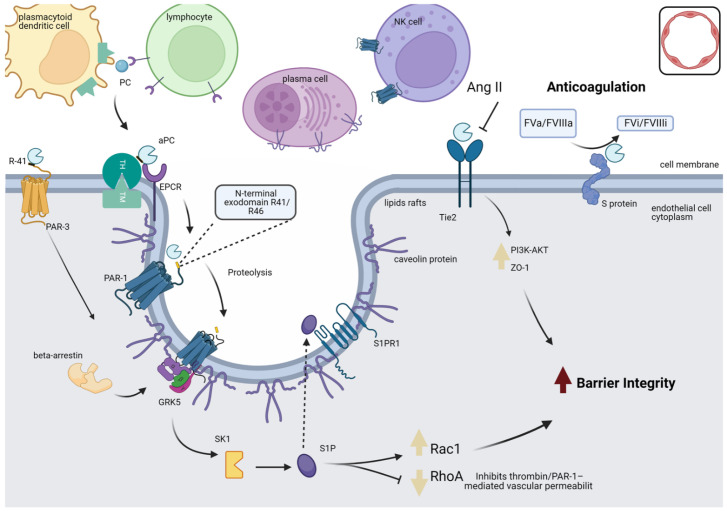
PC activation and signaling in the endothelial cell. When thrombin binds to TM on endothelial cell surfaces, PC is converted to its active form (APC), and EPCR is necessary for optimal PC activation. The co-localization of PAR-1 and EPCR in caveolin-1-rich membrane microdomains specialized in the cell membrane is essential for PAR-1-mediated cytoprotective APC signaling. Furthermore, the heterogeneous expression of PC activation receptors (EPCR, THBD, CAV-1, and PAR-1) in other cell populations suggests that PC activation might occur dynamically by zymogen PC interacting with ‘migratory’ receptors expressed on the surfaces of infiltrating immune cells in the injury site. The anti-inflammatory and anticoagulant actions of APC occur mainly through PAR-1, which, when activated, propagates pro-inflammatory and anti-inflammatory responses according to the protease that cleaved it. PAR-1 is sensitive to proteolysis at its N-terminal exodomain by multiple plasma proteases, including thrombin, APC, plasmin, factor VIIa, factor Xa, and matrix metalloproteases. Cleavage of PAR-1 at the R41/46 sites at the N-terminal exodomain by APC initiates signaling, promoting structural reorganization of the receptor and coupling to the G protein complex (GRK-5) to phosphorylate the intracellular region of PAR-1 and recruitment of β-arrestin 2, which serves as a framework for cytoprotective signaling. This cascade initiates a series of intracellular signaling pathways, including upregulation of SPHK1 activity and, therefore, S1P production, leading to activation of signaling through its S1PR1 receptor, which stimulates a marked increase in Rac1 activation to perform a protective barrier function by stabilizing the endothelial cytoskeleton and inhibiting RhoA signaling. The Tie2 receptor transmembrane domain can initiate downstream signaling pathways and maintain vascular integrity through direct APC binding, which results in increased ZO-1 phosphorylation of Akt via PI3K and ERK inhibition, contributing to increasing the integrity of the barrier. Protein S cofactor-linked APC can intervene at various points during the systemic response to infection. It exerts an antithrombotic effect by inactivating factors Va and VIIIa, limiting thrombin generation. Finally, PAR-3 cleavage by APC can initiate PAR1-dependent cytoprotective signaling in endothelial cells in the presence of EPCR. APC: activated protein C; EPCR: endothelial protein receptor; ERK: extracellular signal-regulated protein kinase; PAR-1: protease-activated receptor 1; PAR-3: protease-activated receptor 3; PC: protein C; PI3K: phosphoinositide 3-kinase; S1P: sphingosine-1-phosphate; SPHK1: sphingosine kinase-1; TH: thrombin; Tie2: transmembrane receptor tyrosine kinase; TM: thrombomodulin.

In addition to decreased *PROC and PROCR* expression, we identified heterogeneous expression of genes associated with PC activators in immune cells. This finding suggests an important aspect of endogenous PC activation in COVID-19 pathogenesis. We know that circulating zymogen PC is activated by interacting with the thrombin-thrombomodulin complex in the presence of EPCR expressed on the surfaces of endothelial and epithelial cells [71,73]. However, our analysis revealed that different immune cells expressed distinct PC activation receptors, suggesting that PC activation might occur dynamically by zymogen PC interacting with ‘migratory’ receptors expressed on the surfaces of infiltrating immune cells in the injury site (Figure 5). PC activation might not be restricted to endothelial cells; it could also be extended to an ‘on-demand extra-vessel activation’ in an immune-mediated PC activation process. These findings highlight the importance of studying the expression of *PROC* and PC activation-related receptors in different cellular subsets.

## 5. Conclusions

Considering the fact that thromboembolic complications are commonly observed in patients with severe COVID-19, as well as our findings showing that the expression of APC signaling-related genes is downregulated in patients with COVID-19, we propose that APC dysfunction in severe COVID-19 could be involved in the development of COVID-19-associated coagulopathy. In addition, considering that *THBD*, *PROCR,* and *F2R* are also involved in the regulation of the inflammatory response [74,75,76], we suggest another important interface that supports the involvement of APC in the pathogenesis of the COVID-19-associated coagulopathy. Furthermore, when visualizing the expression of PC activation-related components and *PROC* in different cell types, we did not identify co-expression of these components, suggesting that PC activation might occur dynamically through the migration and interaction of these circulating cells at the site of the injured tissue. It is possible that SARS-CoV-2 replication occurs in hepatocytes due to their *ACE2* expression, and this replication reprogrammes the hepatocyte transcriptome and affects *PROC* expression. These changes could then contribute to decreased PC synthesis; however, experimental studies are necessary to validate these hypotheses.

There are a few limitations to this study. We used an observational approach and thus could not draw conclusions about causative mechanisms. In addition, we only examined alterations in gene expression. It would be interesting to observe whether there are changes at the protein level.

## Figures and Tables

**Table 1:** Differentially expressed genes related to protein C (PC) activation and signaling in samples from patients with coronavirus disease 2019 (COVID-19). Differentially expressed genes between patients with COVID-19 and healthy controls were determined with Scanpy’s implementation of the two-sided Wilcoxon rank-sum test. Genes lacking a value (denoted as ‘-’) did not reach the logFC threshold of 0.1. COVID-19%: percentage of cells in the COVID-19 group that expresses the gene. Healthy%: percentage of cells in the healthy group that expresses the gene. Abbreviations—logFC: log fold change; PBMC: peripheral blood mononuclear cells.

		Liver				PBMC				Nasal Epithelium			
Gene	Protein Product	LogFC	COVID-19%	Healthy %	Adj. *p*-Value	LogFC	COVID-19%	Healthy %	Adj. *p*-Value	LogFC	COVID-19%	Healthy %	Adj. *p*-Value
*ACE2*	Angiotensin-converting enzyme 2	2.868	0.434	0.068	1.54 × 10^−14^	2.943	0.046	0.006	0.013	0.429	3.336	2.583	0.000161
*AGTR1*	Angiotensin II receptor type 1	1.937	6.534	1.862	2.349 × 10^−98^	-	-	-	-	-	-	-	-
*BAX*	BCL2-associated X protein	−4.150	0.958	14.268	2.285 × 10^−168^	-	-	-	-	0.211	6.778	5.587	0.009326
*BCL2*	BCL2 apoptosis regulator	2.11	12.903	4.323	7.662 × 10^−231^	0.211	20.762	19.053	8.444 × 10^−10^	-	-	-	-
*CAV1*	Caveolin 1	−27.18	0	6.134	2.662 × 10^−80^	2.685	0.889	0.144	3.332 × 10^−31^	-	-	-	-
*F2*	Prothrombin	−1.685	12.572	29.613	1.753 × 10^−167^	-	-	-	-	-	-	-	
*F2R*	Coagulation factor II Thrombin receptor	−2.533	1.751	9.005	9.583 × 10^−78^	−1.127	7.090	14.326	1.136 × 10^−118^	-	-	-	-
*F3*	Tissue factor	1.227	0.722	0.324	3.121 × 10^−06^	1.309	0.249	0.102	0.0004	-	-	-	-
*ICAM1*	Intercellular adhesion molecule 1	−0.284	3.527	4.716		0.906	3.637	1.948	9.690 × 10^−26^	0.548	7.868	4.905	0.000189
*NFKB1*	Nuclear factor kappa B subunit 1	1.812	16.648	6.015	9.235 × 10^−209^	0.121	16.039	14.650	0.0024	−0.261	13.716	16.128	5.76 × 10^−05^
*PROC*	Protein C	−2.192	5.487	19.480	9.039 × 10^−149^	−1.040	0.085	0.174	0.0462	−0.502	1.659	2.432	0.000246
*PROCR*	Endothelial protein C receptor	0.447	1.931	2.376	0.0012	−1.097	0.302	0.643	5.658 × 10^−06^	-	-	-	-
*PROS1*	Protein S	30.593	18.412	0.16	1.731 × 10^−08^	1.026	0.398	0.198	0.00034	−0.985	6.894	12.993	7.8006 × 10^−34^
*RAC1*	Rac family small GTPase 1	−30.680	0	42.942	0	0.259	28.828	24.845	4.315 × 10^−21^	−0.767	34.803	38.897	3.00078 × 10^−33^
*RHOA*	Rho family of small GTPases,	−30.570	0	41.165	0	0.113	54.260	51.530	7.641 × 10^−09^	−0.841	42.300	46.841	1.98516 × 10^−36^
*S1PR1*	Sphingosine-1-phosphate receptor 1	−2.686	0.685	4.306	3.276 × 10^−39^	-	-	-	-	−1.108	0.132	0.254	0.024404
*SPHK1*	Sphingosine kinase 1	-	-	-	-	1.194	2.030	0.872	7.036 × 10^−23^	-	-	-	-
*TEK*	TEK receptor Tyrosine kinase	1.083	5.735	3.093	4.331 × 10^−32^	-	-	-	-	-	-	-	-
*THBD*	Thrombomodulin	−1.636	0.597	1.828	3.007 × 10^−11^	1.881	2.392	0.661	3.457 × 10^−52^	−1.323	0.907	1.605	1.18302 × 10^−07^
*TP53*	Tumor protein P53	−1.013	1.926	4.272	1.501 × 10^−13^	−0.193	6.076	6.850	0.00198	−0.207	4.205	4.719	0.036667

## Data Availability

Data will be available upon request.

## Supplementary Materials

The following supporting information can be downloaded at: https://www.mdpi.com/article/10.3390/v14122753/s1, Table S1: Liver DE genes used to annotate cluster identity; Table S2: PBMC DE genes used to annotate cluster identity; Table S3: Nasal Epithelia DE genes used to annotate cluster identity.
